# Evaluating the efficacy of probiotics in treating Parkinson’s disease model rats using magnetic resonance enhanced gradient echo T2-weighted angiography sequence

**DOI:** 10.3389/fnins.2025.1662530

**Published:** 2025-10-01

**Authors:** Yanmin Su, Yanchao Dong, Jingtao Feng, Xiaoxia Zhang, Ying Yu, Hongzhi Lu

**Affiliations:** ^1^Department of Infectious Diseases, First Hospital of Qinhuangdao, Qinhuangdao, Hebei, PR China; ^2^Department of Medical Imaging Center, First Hospital of Qinhuangdao, Qinhuangdao, Hebei, PR China; ^3^Department of Internal Medicine/Hospital Administrator, Qinhuangdao Maternity and Child Health Hospital, Hebei, PR China

**Keywords:** Parkinson’s disease, probiotics, ESWAN sequence, substantia nigra, iron deposition, gut–brain axis

## Abstract

**Background:**

Parkinson’s disease (PD) involves iron deposition in the substantia nigra (SN) and loss of dopaminergic neurons, with gut microbiota dysbiosis potentially affecting the brain iron via the gut–brain axis, whereas magnetic resonance enhanced gradient echo T2-weighted angiography (ESWAN) enables non-invasive iron assessment. Currently, for the detection of brain iron content, traditional tissue biopsy can provide accurate pathological information. However, its clinical and scientific research applications are limited owing to its invasiveness, difficulty in repeated operations, and other limitations. This study aimed to evaluate the therapeutic efficacy of probiotics and underlying mechanisms in PD rats using ESWAN.

**Methods:**

Twenty-seven male Sprague–Dawley rats were divided into control, PD model, and probiotic treatment groups. PD models were established by 6-hydroxydopamine stereotaxic injection into the SN, whereas the treatment group received oral gavage of *Lactobacillus acidophilus* and *Bifidobacterium bifidum* mixture. ESWAN was used to quantify iron deposition, complemented by iron histochemical staining and wire grip tests for motor function assessment.

**Results:**

The probiotic treatment significantly reduced right SN R2* values (*p* < 0.0001) and iron staining optical density (*p* < 0.0001), and prolonged the time of wire grip duration (*p* < 0.0001) compared with PD rats, though not fully to control levels. Mechanistically, probiotics likely alleviated iron overload via gut–brain axis modulation, antioxidant enzyme enhancement, and blood–brain barrier maintenance. The strong positive correlation (*r* ≥ 0.7) between ESWAN-derived R2* values and iron staining confirmed ESWAN as a reliable non-invasive tool for brain iron assessment.

**Conclusion:**

This study provides experimental evidence that probiotics mitigate SN iron deposition in PD rats by regulating gut microbiota, highlighting ESWAN as a potential imaging biomarker for early PD diagnosis and therapeutic monitoring.

## 1 Introduction

Parkinson’s disease (PD) is a common neurodegenerative disease that predominantly affects middle-aged and older adults. Its most characteristic pathological feature is the progressive loss of dopaminergic neurons in the substantia nigra (SN). Abnormal iron deposition in the SN is an important pathological change in PD; however, its underlying mechanism remains unclear. Iron serves as an indispensable cofactor in neuronal metabolic processes (Lee and Roh, 2023; Rogoz et al., 2024; Wang et al., 2021). Excess iron or iron overload can lead to the death of dopaminergic neurons in the SN through multiple pathways, such as inducing mitochondrial dysfunction and promoting iron-dependent cell death (Zeng et al., 2024).

Reportedly, significant changes in gut microbiota, such as alterations in the abundance of Bifidobacteriaceae and Christensenellaceae (Chiang and Lin, 2019), often occur in PD patients. Gut microbiota dysbiosis affects the local intestinal environment and influences brain health through multiple pathways. Transplantation of intestinal microbiota from patients with PD into wild-type mice can lead to intestinal inflammation and α-synuclein aggregation in mice, thereby triggering damage to dopaminergic neurons and mitochondrial dysfunction in the midbrain SN region (AlRuwaili et al., 2025; Li et al., 2023). A potential link is observed between the gut microbiota and iron metabolism. Gut microbiota can regulate plasma proline levels, affecting brain iron deposition (Yang et al., 2024). This suggests that modulating the gut microbiota may have an impact on brain iron deposition.

The gut microbiota can convert levodopa into dopamine via the tyrosine decarboxylase pathway and further degrade it into m-tyramine, thereby reducing the bioavailability of the drug (Menozzi and Schapira, 2024). Meanwhile, intestinal microbiota disorders commonly observed in PD patients, such as small intestinal bacterial overgrowth and Helicobacter pylori infection, can also affect levodopa metabolism (Menozzi and Schapira, 2024). Additionally, intestinal dysbiosis can increase the permeability of the blood-brain barrier and blood-cerebrospinal fluid barrier, enabling microbiota metabolites to enter the brain and promote neurodegeneration (Kapoor et al., 2024; Xie J. et al., 2023; Xie Z. et al., 2023). Interventions targeting the gut microbiota (e.g., regulating microbiota composition through the Mediterranean diet, inhibiting bacterial decarboxylase activity) may serve as new strategies to improve motor complications and levodopa-unresponsive symptoms in PD (Zhang et al., 2025).

Currently, for the detection of brain iron content, traditional tissue biopsy can provide accurate pathological information; However, its clinical and scientific research applications are limited owing to its invasiveness, difficulty in repeated operations, and other limitations (Simuni et al., 2024; Vijiaratnam and Foltynie, 2023; Yan et al., 2024). The development of magnetic resonance imaging (MRI) technology has provided a new approach for non-invasive assessment of brain structural and metabolic changes. Among them, the enhanced gradient echo T2-weighted angiography (ESWAN) sequence is considered a mature method in magnetic resonance technology for evaluating iron content changes in the SN (Wang et al., 2013). Literature reports that the R2* value measured by ESWAN has a significant positive correlation with brain iron content (Hartono et al., 2023). Through sensitive detection of local magnetic field inhomogeneity, it can accurately reflect the degree of iron deposition in tissues (Alushaj et al., 2024). As a non-invasive examination method, ESWAN has the advantages of safety, repeatability, and easy access. It can avoid irreversible damage to experimental animals and realize longitudinal dynamic observation of the same subject, providing an ideal imaging method for evaluating the efficacy of probiotics in treating PD rats (Ji et al., 2016; Ren et al., 2024). In-depth exploration of the application of the ESWAN sequence in the efficacy evaluation of probiotics in treating PD rats is expected to reveal a new path for non-invasive diagnosis of PD and evaluation of treatment effects, and also provide a certain theoretical basis for clinicians to transform probiotic treatment strategies for PD from basic research to clinical practice. This study aimed to evaluate the therapeutic efficacy of probiotics and underlying mechanisms in PD rats using ESWAN.

## 2 Materials and methods

### 2.1 Animals

Twenty-seven Sprague–Dawley rats (male, 7 weeks old, and weighing 270 ± 10 g) were obtained from Beijing Vital River Laboratory Animal Technology Co., Ltd., China. The experiments were conducted in accordance with national guidelines for the use of experimental animals. All experimental protocols were approved by First Hospital of Qinhuangdao ethics committee (202401A221). The animals were housed (one animal per cage) in a controlled environment under a 12-h dark and 12-h light cycle, temperature range of 22–24°C, and relative humidity of 60 ± 5% at the Laboratory Animal Center of Yanshan University (Qinhuangdao city, Hebei province, China). Water and food were provided *ad libitum*.

### 2.2 Study design

Rats were randomly divided into treatment groups (*n* = 9), PD (*n* = 9), and control group (*n* = 9). The sample size of 9 animals per group is based on calculations using G*Power software (Faul et al., 2009). The calculation parameters were as follows: Effect size *f* = 0.8 (previous research indicated significant differences in mean values between groups), α err prob = 0.05, Power (1-β err prob) = 0.8, and Number of groups = 3. The results showed that a sample size of 8 animals per group would be sufficient to detect statistical differences. To account for potential attrition, we chose 9 animals per group to ensure the validity of the final analysis. To reduce bias, the present study employed the following randomization and blinding procedures: Animals were randomly assigned to 3 groups (9 animals per group) using a random number table generated via Excel, with the allocation conducted by an independent researcher not involved in subsequent experiments. All the remaining 18 rats underwent the modeling process except for the rats in the control group. The modeling procedures are as follows: first, the rat was anesthetized using 10% chloral hydrate by intraperitoneal injection. The initial measurement was 3 mL/kg, and the maintenance dose was 2 mL/kg/h for the duration of the surgery. The rat was mounted into a stereotactic apparatus (Lab Standard Stereotaxic-Single, Stoelting Co, Illinois, United States). Bregma was exposed. An incision was made on the scalp along the sagittal suture, and a small trephine hole was drilled in accordance with the stereotactic coordinates of the right SN (relative to bregma: anterior-posterior, −4.8 mm; medial-lateral, 1.9 mm; dorsal-ventral, −8.0 mm from skull). We chose coordinate points according to George Paxinos’ book, the Rat Brain in Stereotaxic Coordinates, Compact, Third Edition. Using a microsyringe (Hamilton Bonaduz AG, Bonaduz, Switzerland), 6 μL of 6-hydroxydopamine solution (2 μg/μL in normal saline containing 0.2% ascorbate; Sigma Chemical Co., St. Louis, MO, United States) was automatically infused into the right SN of rats in the two groups. Drugs can also cause serious damage to noradrenergic neurons; however, we did not protect noradrenergic neurons before injecting 6-hydroxydopamine (Lindgren et al., 2014). The needle was kept in place for 5 min after completion of the injection and then slowly withdrawn. The scalp was sutured closed, and intramuscular antibiotics were administered to prevent infection. Rats in the control group were injected with the same volume of 0.2% ascorbic acid. All rats should be observed daily from the time they regain consciousness after intraperitoneal injection anesthesia until euthanasia. The observation contents include behavioral changes (such as the presence or absence of reduced activity levels, lethargy, and fluffy and disheveled fur), as well as abdominal signs at the injection site (such as the presence or absence of abdominal swelling, skin redness and swelling, local tenderness, and local hair loss caused by frequent licking of the puncture site due to discomfort). Until the end of the experiment, none of the rats that had undergone intraperitoneal injection anesthesia showed the aforementioned peritonitis symptoms or signs.

All outcome measures (e.g., motor function tests, detection of brain iron deposition) were assessed by researchers who were blinded to the animals’ group assignments and treatment status. During the assessment, animals were identified only by numerical codes, and the codes were not decrypted until the completion of data analysis.

### 2.3 Apomorphine-induced rotation test

In the 2*^nd^* week after modeling, PD and treatment group rats received a single intraperitoneal injection of apomorphine (0.5 mg/kg in normal saline) at the beginning of every week after surgery. For the rotation test, animals were allowed to habituate to the test apparatus for 10 min and then for an additional 2 min after the injection. Full rotations were counted in a cylindrical container in a dimly lit, quiet room. Rotational asymmetry was scored continuously for 30 min; then the complete contralateral rotation times were scored. Rats with test scores greater than 7 were retained for the study and analysis.

After a 6-hydroxydopamine lesion, we needed to conduct a rotation test to verify the success of the animal model. Currently, the rat model is generally believed to be successfully prepared if the rotation experiment exceeds seven times per minute.

### 2.4 Administration of probiotics

According to previous studies (Westbrook et al., 2014), the powder of *Lactobacillus acidophilus* and *Bifidobacterium bifidum* produced by Xiangfu Biological Co., Ltd. (Shanghai, China) was re-dissolved in distilled water to prepare a solution with a concentration of 2 × 10^9^ colony-forming unit organisms per milliliter (the ratio of two probiotics should be as close as possible to 1:1). After animal grouping, rats in the treatment group, which were PD rats that had passed the apomorphine rotation test, were started on oral gavage with the same probiotic-mixed solution once a day from the 2*^nd^* week after modeling, and this intervention lasted until the end of the 6*^th^* week of the experiment. Rats in the PD and HC groups were administered the same volume of distilled water by gavage.

### 2.5 MRI

MRI was performed respectively before the experiment and at the 6*^th^* week after the experiment. Anesthesia was performed as earlier, and the rats were scanned using a 3.0T MRI system (HD, General Electric Co., Waukesha, Wisconsin, USA) with an eight-channel coil. ESWAN was performed using a three-dimensional multiple gradient echo sequence with slices paralleling to the anterior–posterior commissural line. Imaging parameters were magnetic resonance acquisition Type: three-dimensional, repetition time = 36.8 ms, echo time = 13.8 ms, number of echoes = 8, flip angle = 20°, slice thickness/gap = 1 mm/1 mm, number of excitations = 1, field of view = 5.6 × 7.9 cm^2^, matrix = 256 × 256, number of slices = 304, and total acquisition time = 3 min 14 s.

Two senior radiologists with over 10 years of experience in neural MRI analysis and unaware of the animal groupings referred to the Rat Brain in Stereotaxic Coordinates (3rd Edition). They selected the slice in the MRI images that showed the largest cerebral SN, and manually outlined regions of interest (ROIs) at the center of the right side SN in this slice. The area of these regions ranged from 0.30 to 0.60 cm^2^. The average value of the two repeated measurements of the SN was used for the final analysis. The measurement results of the two radiologists were tested for consistency using the intraclass correlation coefficient (ICC). Then, the measurement results of the two radiologists were averaged and used as the final measured MRI parameters.

### 2.6 Wire grip test

The motor coordination or neurological function of rats was evaluated using the wire grip test. The rats were suspended with both forepaws on a horizontal steel wire (60 cm in length, 7 mm in diameter). When the forepaws of the rats made contact with the wire, they were kept in a vertical suspended position. A stopwatch was used to record the time for the rats to fall off the wire. The rats were tested randomly, and each animal was given one trial. Each rat was trained for the task three times before the formal experiment (Hadadianpour et al., 2017).

### 2.7 Histochemistry

After all rats completed the above experiments, they were euthanized by intraperitoneal injection of pentobarbital sodium at a dose of 100 mg/kg. Pentobarbital sodium was purchased from Sigma-Aldrich (St. Louis, MO, USA). Subsequently, the intact brain tissues of the rats were removed and placed in formaldehyde fixative. Subsequently, for the SN region of the rat brain tissue, steps including fixation, dehydration, clearing, paraffin impregnation, embedding, sectioning, mounting, and deparaffinization-rehydration were performed. A portion of the sections was then selected. The selected sections were subjected to iron hematoxylin histochemical staining according to the method described by Zhang et al. (2014). Sections were processed through various graded alcohols, into xylene, and rehydrated to water. The sections were incubated in a 1:1 solution of 2% hydrochloric acid and potassium ferrocyanide (Sigma-Aldrich, St. Louis, MO, United States) for 30 min and rinsed in water. Then they were counterstained with Neutral Red, dehydrated in increasing concentrations of ethanol, cleared in xylene, and mounted on slides (Zhang et al., 2014).

The intensity of iron-positive staining in tissue sections was analyzed by integrated optical density (IOD) using the Image-Pro Plus 6.0 software (Media Cybernetics, United States) as described previously with minor modification (Wang et al., 2009). Four 20× TIFF-format images from ten individual rats in each group were analyzed in a blinded manner. All the images were captured using the same microscope and camera sets. The Image-pro Plus software was used to calculate the average IOD per stained area (μm^2^) (IOD/area) for positive staining. The IOD function of Image-Pro Plus 6.0 was used with the following settings: preprocessing involved rolling ball background subtraction (radius = 50 pixels) and Gaussian smoothing (σ = 1.0); thresholding was performed using the Otsu algorithm with manual validation to exclude non-specific staining, and positive areas were quantified by particle counting with a size filter (≥50 μm^2^) to remove noise.

### 2.8 Statistical analysis

Statistical analyses were performed using Statistical Package for Social Sciences (Chicago, IL) version 21.0 software. All data were subjected to normality test using the Shapiro-Wilk test, *p* > 0.05 still indicates compliance with the normal distribution. The data conforming to the normal distribution were presented as the mean ± standard deviation. The consistency of the data measured by the two radiologists was evaluated using the ICC, and ICC value greater than 0.75 would be considered to indicate good consistency. Significant between-group differences were evaluated using one-way analysis of variance and least significant difference *post hoc* tests. The comparison of FA of rats in each group at different time points was performed using the repeated measures analysis of variance. Pearson’s correlation analysis was employed to assess the correlation of the IOD/area of glial fibrillary acidic protein and iron staining density with the MRI parameters. Statistical differences were considered significant when *p* < 0.05; |r| ≥ 0.7 was considered a strong correlation.

## 3 Results

### 3.1 Comparison of magnetic resonance parameters in the right SN region of the three groups of rats

The consistency test results for the magnitude and R2* values measured by the two radiologists revealed ICC of 0.998 and 0.972, respectively, indicating good data consistency for both parameters.

**FIGURE 1:**
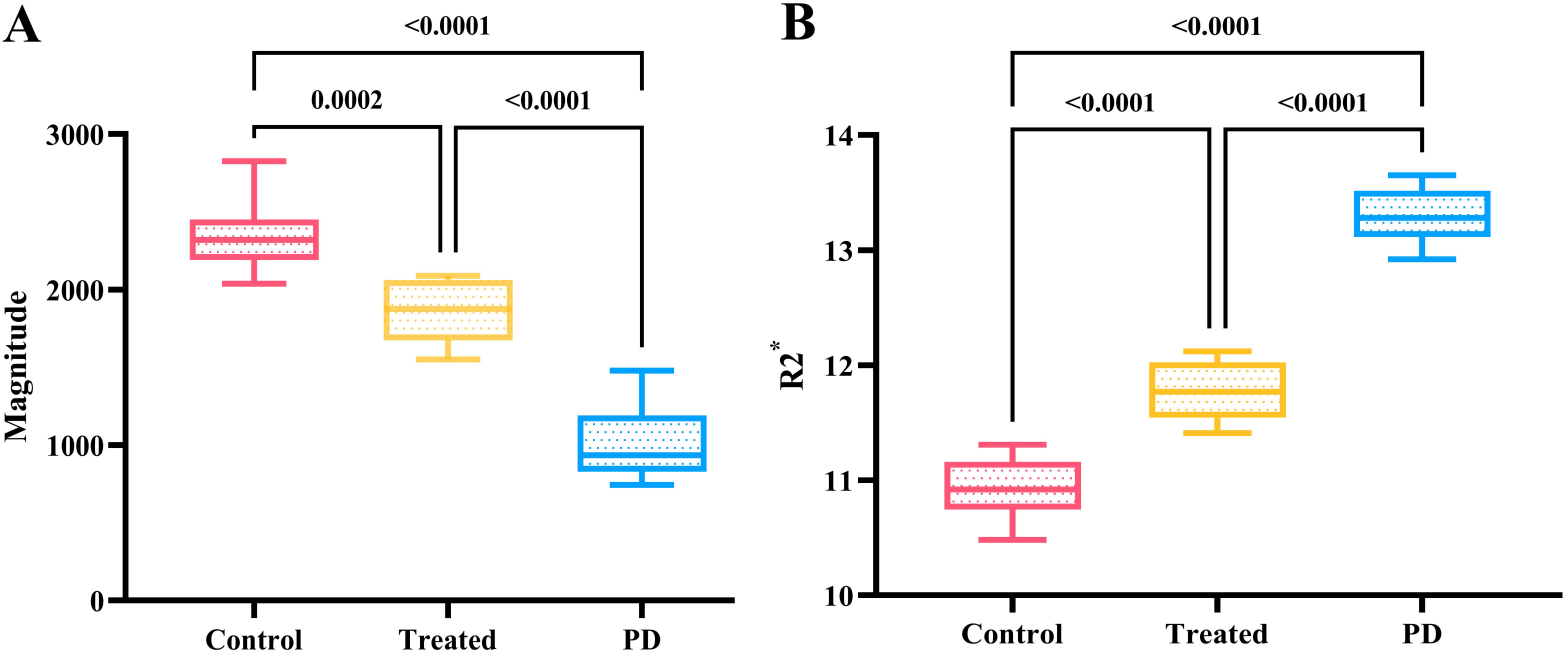
**(A)** Shows the comparison results of magnitude values in the right SN region of three groups of rats at the 6th week. The magnitude value of the control group was the highest, followed by the treatment group, while the PD group had the lowest magnitude value, with statistically significant differences. **(B)** Displays the comparison results of R2* values in the right SN region of the three groups at the 6^th^ week. The control group had the lowest R2* value, followed by the treatment group, while the PD group had the highest R2* value, and the differences were statistically significant.

**FIGURE 2:**
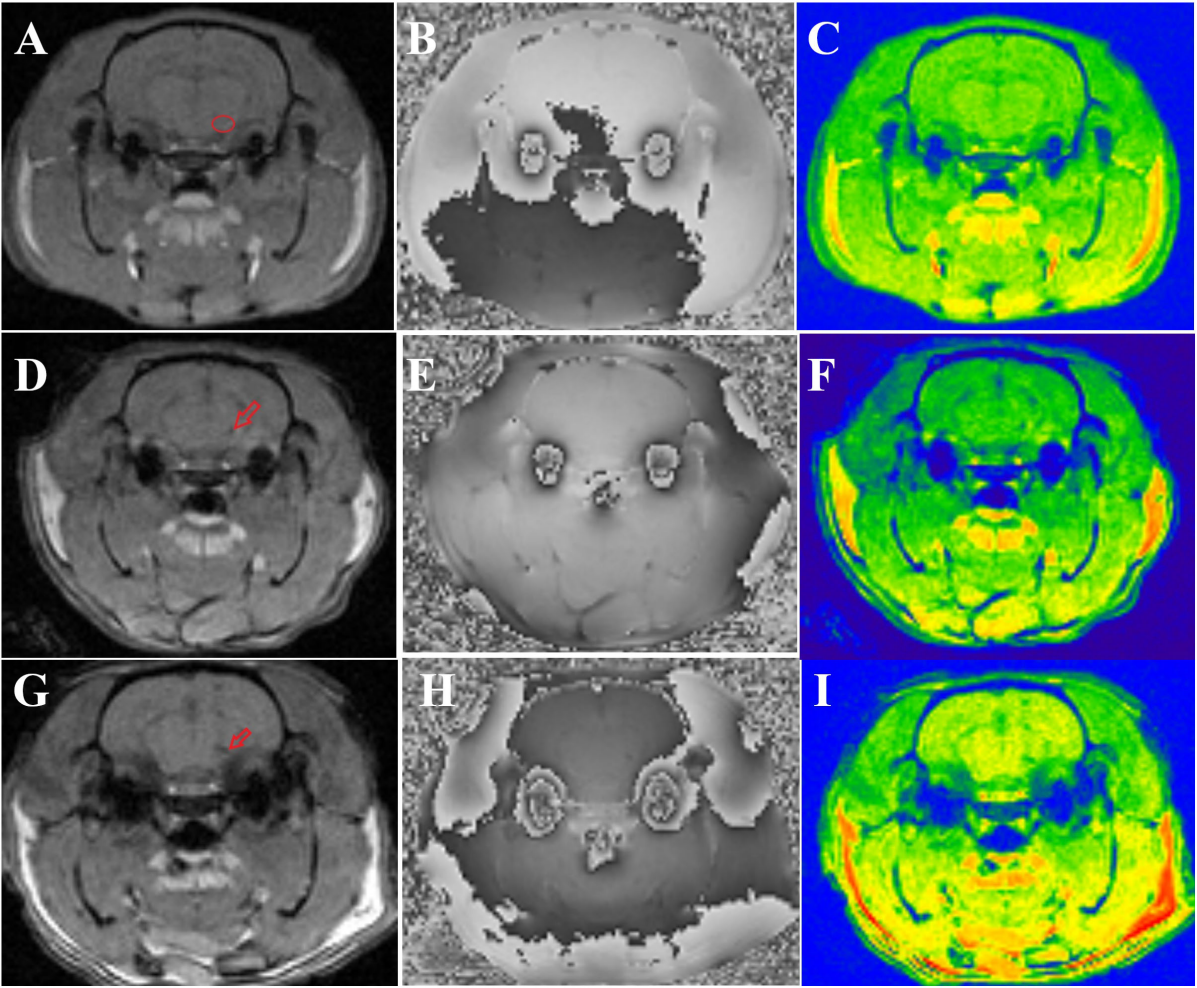
Shows the magnetic resonance images of three groups of rats. **(A–C)** Display the magnitude, phase, and R2* map of control group rats, respectively. The small red circle in panel **(A)** indicates the location of the ROI outlined in the right SN. (**D–F)** show the magnitude, phase, and R2* map of treatment group rats, respectively, with the small red arrow in panel **(D)** indicating iron deposition in the right SN. **(G–I)** present the magnitude, phase, and R2* map of PD group rats, respectively, and the small red arrow in panel **(G)** denotes iron deposition in the right SN.

There were no significant differences in the magnitude or R2* values among the three groups of rats before modeling (*p* > 0.05), see Table 1 for details. At 6*^th^* week, the results showed significant statistical differences in the magnitude values and R2* in the right SN region among the three groups of rats (magnitude: *F* = 82.46, *p* < 0.0001; R2*: *F* = 205.00, *p* < 0.0001). Specifically, the magnitude values in the right SN region of the treatment group were significantly higher than those in the PD group (*p* < 0.0001), whereas the R2* values were significantly lower (*p* < 0.0001), suggesting a reduction in susceptibility in the right SN region after probiotic treatment. However, the magnitude values in the treatment group remained lower than those in the control group (*p* = 0.0002), and the R2* values were significantly higher (*p* < 0.0001), indicating that even with active probiotic treatment, the brain state might not fully return to that of the unmodeled rats (Figure 1). The magnetic resonance images of the SN level in the brains of the three groups of rats are shown in Figure 2.

**TABLE 1:** Shows the comparison results of magnitude or R2* values among the three groups of rats before modeling.

MRI parameters	Group comparison (mean ± SD)	*F*	*P*	LSD-P
Magnitude	Control (2290.44 ± 172.85) vs. Treatment (2194.78 ± 117.97)	2.151	0.127^#^	0.055^#^
	Control (2290.44 ± 172.85) vs. PD (2204.78 ± 105.26)			0.068^#^
	Treatment (2194.78 ± 117.97) vs. PD (2204.78 ± 105.26)			0.784^#^
R2*	Control (10.81 ± 0.36) vs. Treatment (10.83 ± 0.65)	0.804	0.453^#^	0.923^#^
	Control (10.81 ± 0.36) vs. PD (11.01 ± 0.53)			0.340^#^
	Treatment (10.83 ± 0.65) vs. PD (11.01 ± 0.53)			0.282^#^

^#^Represents no statistically difference.

### 3.2 Comparison of the wire grip test among the three groups of rats

**FIGURE 3:**
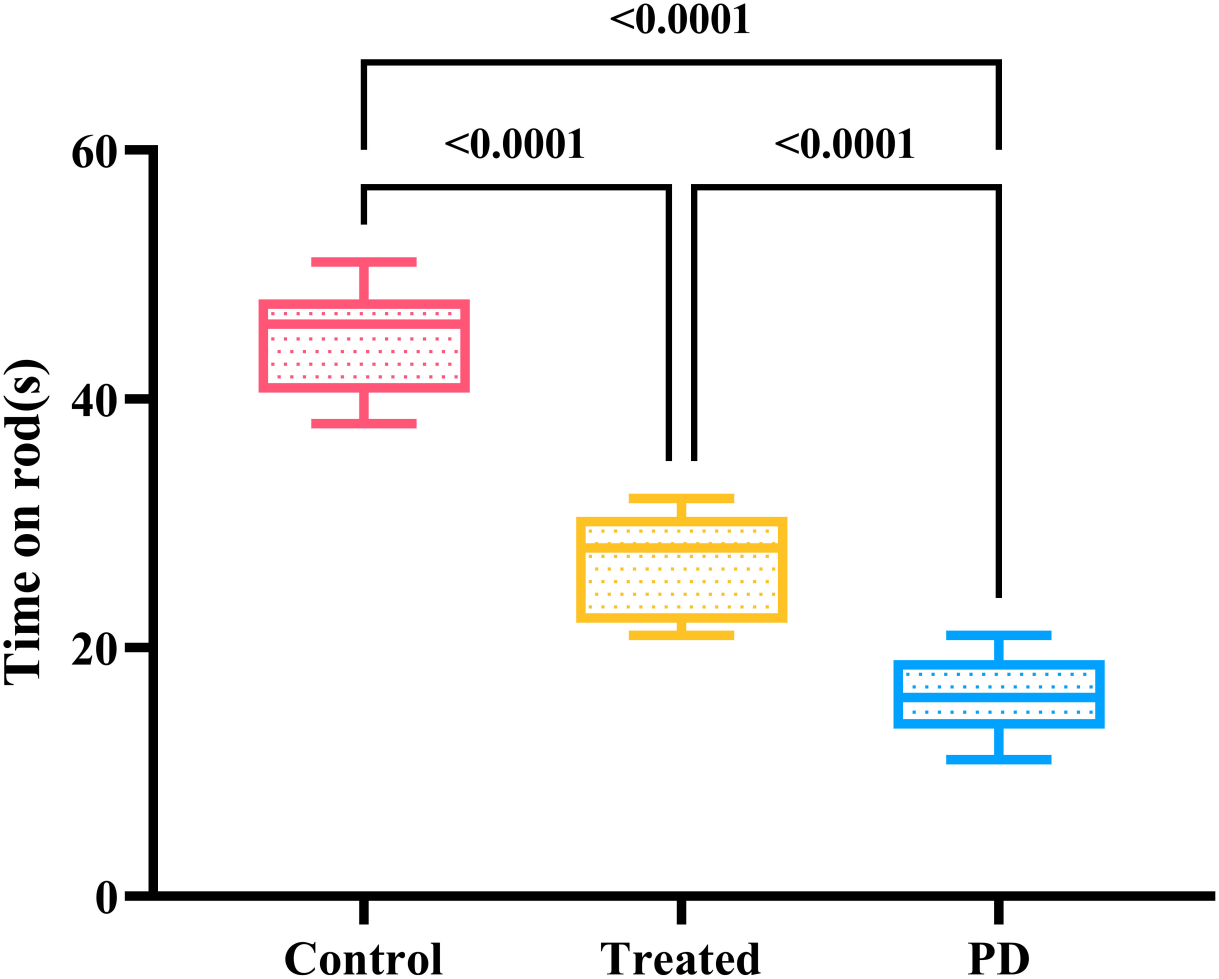
Shows the results of the motor experiment in three groups of rats. The control group spent the longest time on the wire, followed by the treatment group, while the PD rats had the shortest persistence time on the wire. The differences were statistically significant.

Both the Parkinson’s and the treatment group rats passed the apomorphine rotation test. The results of the wire grip test in the three groups of rats showed that the duration of persistence on the wire of PD rats was significantly lower than that of the treatment and control groups, and the difference was statistically significant (*F* = 36.48, *p* < 0.0001). Among them, the persistence time of rats in the treatment group remained lower than that in the control group, which reflects that active probiotic treatment can lead to a certain degree of recovery in the motor ability of rats (Figure 3).

### 3.3 Comparison of IOD/area for iron staining in the right SN region of the three groups of rats

**FIGURE 4:**
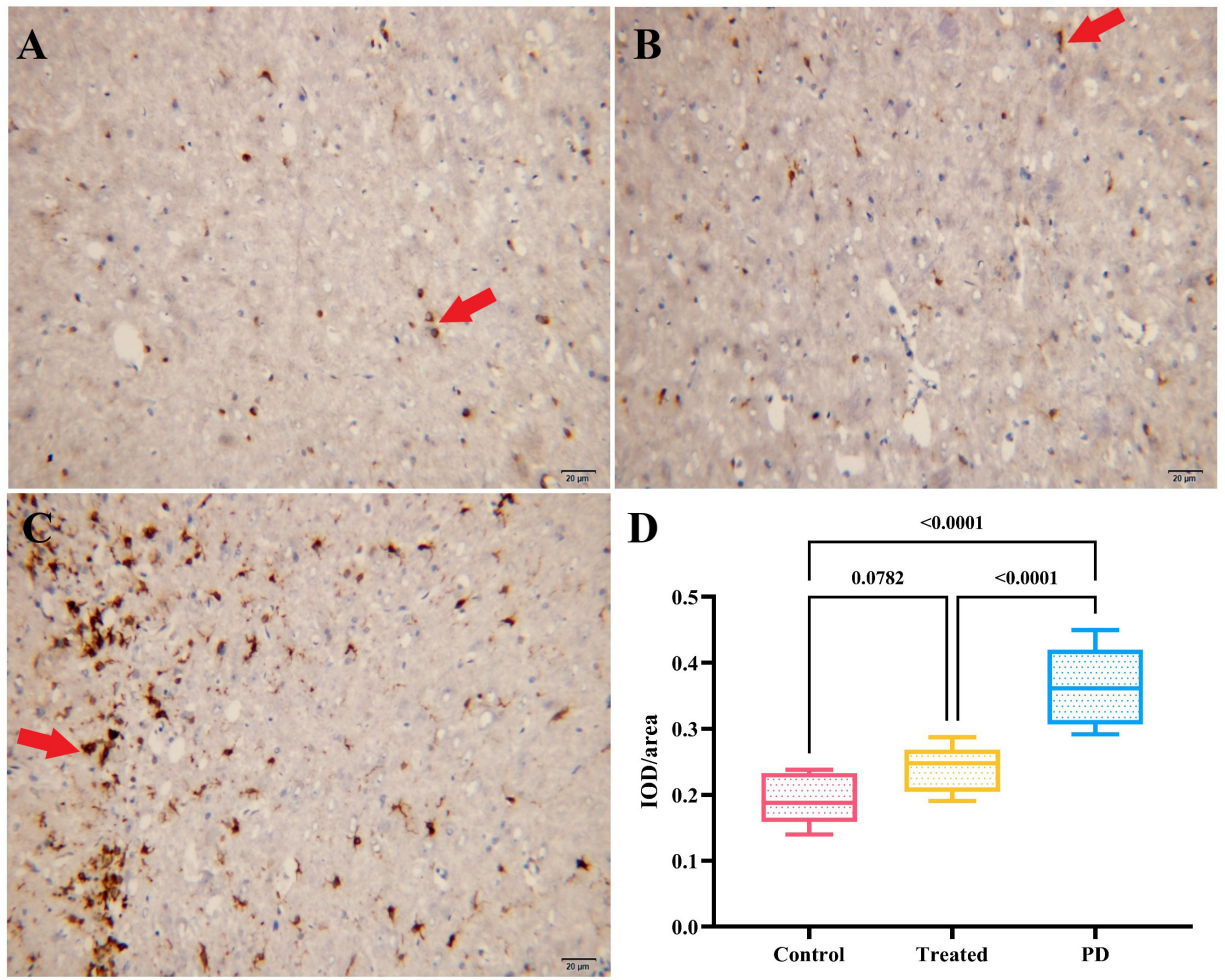
**(A–C)** Show the iron staining results of the right SN region in the control group, treatment group, and PD group rats, respectively. The cells indicated by the red arrows are iron stain-positive cells. **(D)** Displays the comparison of IOD/area values of iron staining in the right SN region among the three groups. The PD group had the highest IOD/area value of iron staining, followed by the treatment group, and the control group had the lowest value.

The results showed that the average optical density values of iron staining in the right SN region of the rats in both the treatment the control groups were significantly lower than those in the PD group, and the differences were statistically significant (*F* = 116.70, *p* < 0.0001). However, no statistical difference as observed in the average optical density values of iron staining in the right SN region between the treatment and the control group rats (*p* = 0.0782). It indicates that active probiotic treatment may significantly reduce the degree of iron deposition in the SN. Figure 4 shows details and the iron staining images of the three groups.

### 3.4 Correlation analysis among magnetic resonance parameters, motor experiments, and immunohistochemical data

**FIGURE 5:**
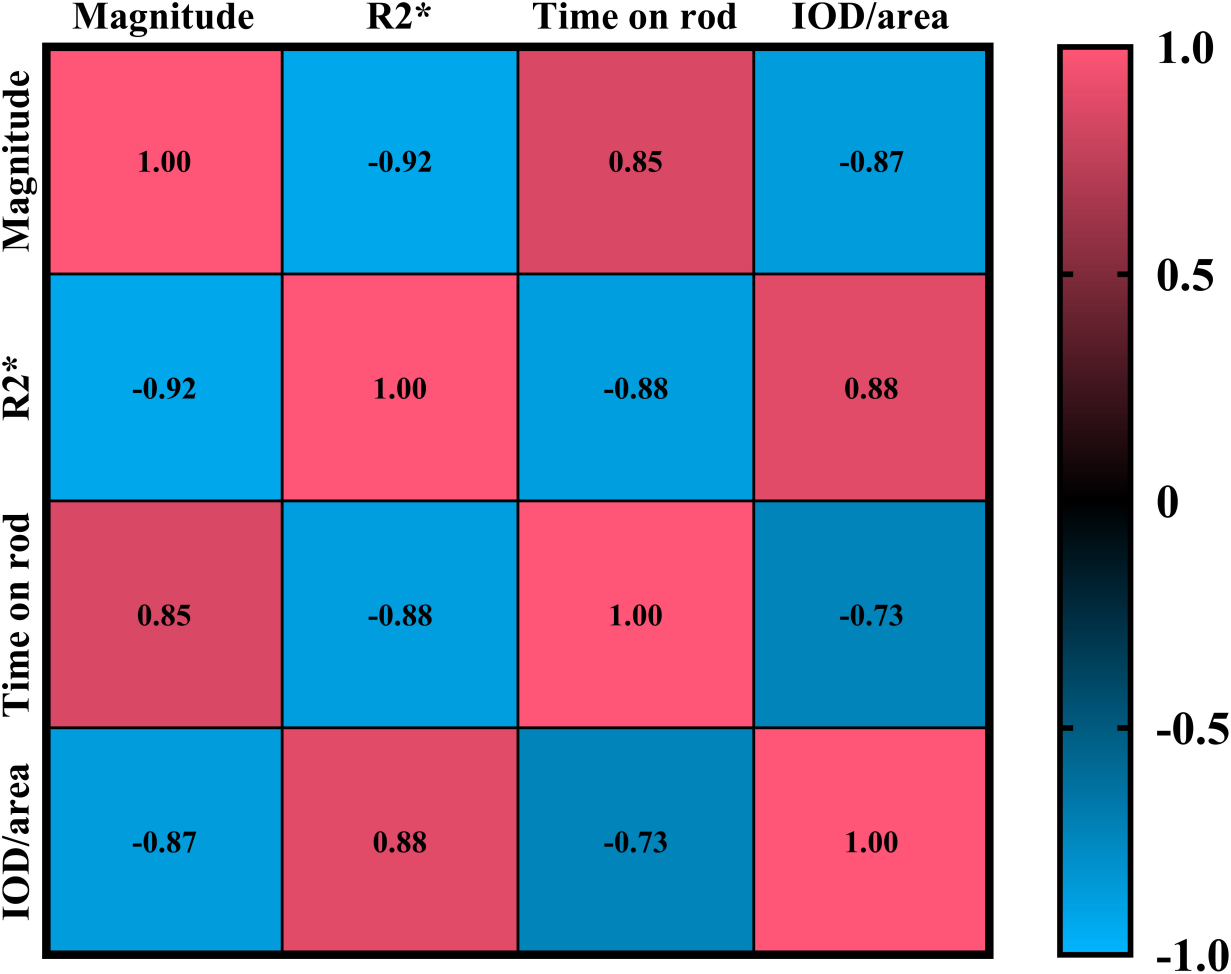
Shows the Pearson correlation results of Magnitude, R2*, time on rod, and IOD/area. In the figure, red indicates a positive correlation, and blue indicates a negative correlation. The numbers in the figure represent correlation coefficients, with all *p* < 0.0001.

The results showed that the magnetic resonance magnitude values were strongly negatively correlated with both the R2* values and the average optical density values of iron staining positivity (Magnitude vs. R2*: *p* < 0.0001, CI = −0.9646 to −0.8361; Magnitude vs. IOD/area: *p* < 0.0001, CI = −0.9372 to −0.7231). In contrast, they were strongly positively correlated with the duration of persistence on the wire in the motor experiment (*p* < 0.0001, CI = 0.6937 to 0.9296). For details, please refer to Figure 5.

## 4 Discussion

### 4.1 Effects of probiotic treatment on iron deposition in the SN of PD rats

The rationale for selecting these two probiotics in this experiment is as follows: the combined use of probiotics containing *Lactobacillus acidophilus* and *Bifidobacterium bifidum* has been employed in clinical studies to improve intestinal and nervous system-related diseases, suggesting their potential neuroprotective value (Klingenberg et al., 2025). The underlying mechanism may be that these two strains can promote the production of short-chain fatty acids (SCFAs) (such as acetic acid, butyric acid, and lactic acid) (Hu et al., 2021; Pan et al., 2022), and these metabolites affect neuroinflammation and oxidative stress through the gut-brain axis (Aytekin Sahin et al., 2022; Taghizadeh Ghassab et al., 2024). In addition, in studies on PD and Alzheimer’s disease (AD) models, the probiotic combination containing these two strains has been shown to reduce nerve damage, Aβ pathology, and neuroinflammation. It can also improve memory impairment and influence neuropathology by regulating serum short-chain fatty acid levels (Flynn et al., 2025; Xiao-Hang et al., 2024). To sum up, the selection of these two strains is mainly based on their direct neuroprotective effects, ability to regulate the gut-brain axis, antioxidant/anti-inflammatory properties, and validated results in various neurological disease models.

This study confirmed that probiotic treatment significantly reduced iron deposition in the SN of PD rats. The mean optical density value of iron staining in the SN of the treatment group significantly decreased compared with that in the PD group (*p* < 0.0001), and was consistent with the decreasing trend of R2* values measured by the ESWAN sequence, suggesting that probiotics can alleviate SN iron overload by regulating the gut microbiota. The possible mechanisms of this finding are as follows: (1) Probiotics may promote dephosphorylation by enhancing alkaline phosphatase activity, potentially reducing phosphate levels, which could in turn lead to decreased brain iron deposition in mouse models, and might ultimately contribute to improvements in cognitive dysfunction in mouse models with a high-iron diet (Zhang Y. et al., 2024); (2) Probiotics might regulate gut-brain axis interactions, could potentially affect the expression differences of iron transport transport-related genes (such as divalent metal transporter 1, transferrin receptor protein 1), and might simultaneously contribute to improving the correlation between gut microbiota imbalance (such as increasing Butyricimonas and decreasing Paraclostridium) and cognitive function (Suparan et al., 2024; Zhang Y. et al., 2024); (3) Probiotics might conceivably mitigate secondary cerebral tissue damage induced by systemic iron overload, presumably through modulation of the expression of hepatic iron storage protein ferroportin 1 and key inflammatory markers (nuclear factor kappa-B, tumor necrosis factor-α) (Shete et al., 2024).

Probiotics, on the contrary, regulate the gut microbiota by promoting the production of SCFAs. These SCFAs, such as acetate, propionate, and butyrate, are the main metabolites produced by the fermentation of dietary fiber by intestinal microorganisms (Ikeda et al., 2022; Xie L. et al., 2023). They act as an energy source to maintain colon health and protect the host from diseases such as inflammatory bowel disease through anti-inflammatory and immunomodulatory functions (Mirzaei et al., 2022; Yan et al., 2023; Zhang et al., 2022). SCFAs improve the composition of the gut microbiota by increasing the abundance of beneficial bacteria (such as Bacteroidetes and Actinobacteria) while reducing the number of pathogenic bacteria (such as Fusobacteria and Megamonas) (Wang et al., 2021; Zhang L. et al., 2024). Additionally, as signaling molecules, SCFAs enter the systemic circulation and affect metabolic, immune, and nervous system functions, including regulating intestinal barrier function, immune cell development, and gut–brain axis communication (Cao et al., 2025; Golpour et al., 2023; Xie et al., 2025). Moreover, SCFAs can establish communication between the enteric nervous system and the central nervous system through the vagus nerve, immune circulation, and endocrine signaling (Pang and Ren, 2026). Probiotic intervention can further alleviate dysbiosis and related diseases by restoring the abundance of SCFA-producing bacteria (such as Roseburia and Faecalibacterium) (Crawford et al., 2024; Jin et al., 2022).

These effects may be related to mechanisms such as maintaining blood–brain barrier integrity (Yuan et al., 2024), inhibiting microglial activation (Carota et al., 2022), and regulating mitochondrial dynamics (Sripetchwandee et al., 2024). However, the degree of iron deposition in the treatment group remained higher than that in the control group (*p* = 0.0002), indicating that probiotics can partially reverse the pathological process; however, they cannot completely restore normal iron metabolism, which may be related to the irreversible neuronal damage existing in the PD model. Probiotic treatment inhibits further neuronal loss to a certain extent through these mechanisms; however, it cannot completely prevent the loss of dopaminergic neurons.

### 4.2 Diagnostic value of magnetic resonance ESWAN sequence

The ESWAN sequence demonstrated excellent quantitative ability for iron deposition in this study. The measured R2* value showed a strong positive correlation with the optical density of iron staining (*r* ≥ 0.7), confirming that this technique can serve as a reliable tool for non-invasively evaluating brain iron content. ESWAN can quantify iron content in nuclei such as the SN, and its measurement results are related to the severity of clinical symptoms, significantly increasing the area under the curve value for early PD diagnosis to 0.904 (Alushaj et al., 2024; Yan et al., 2024). Compared with traditional tissue biopsy, ESWAN has the advantage of repeatability, enabling dynamic monitoring of the same animal, which is crucial for evaluating the dynamic changes in probiotic efficacy. Additionally, this study observed that the R2* value in the SN of PD rats significantly increased while the magnitude value decreased, which is consistent with the imaging characteristics of clinical patients with PD, suggesting that ESWAN may be used for preclinical screening of PD. As iron deposition precedes the loss of dopaminergic neurons (Zhou et al., 2024), it may become a biomarker for early disease diagnosis.

As an iron-sensitive MRI technique, ESWAN boasts unique advantages and innovativeness compared with SWI (Susceptibility-Weighted Imaging) and QSM (Quantitative Susceptibility Mapping). SWI, sensitive to paramagnetic substances like iron via combining amplitude and phase information, cannot directly quantify susceptibility or iron concentration (only indirectly reflecting iron deposition through signal intensity changes); its phase data also require high-pass filtering, which may lose low-frequency field information and affect accurate assessment of iron deposition in deep brain regions (e.g., basal ganglia) (Lu et al., 2022). QSM provides absolute susceptibility values by solving the field-susceptibility inverse problem but faces challenges: complex post-processing (e.g., background field removal, dipole inversion) susceptible to phase noise and algorithm selection, and lack of wide standardization leading to significant inter-institutional reconstruction differences (Gao and Liang, 2025; Harada and Kudo, 2022). ESWAN uses a multi-echo gradient echo sequence to obtain T2* decay curves, achieving more direct iron quantification by fitting and calculating R2* (1/T2*) values (Reeder and Yokoo, 2023). There were studies show ESWAN R2* values are highly correlated with histological iron concentration (e.g., in neurodegenerative diseases) (Guan et al., 2021; Ji et al., 2016). It works on conventional 1.5T/3T MRI without ultra-high field strength (7T), reducing clinical application thresholds (Hernando and Zhao, 2023). In iron quantitative applications, ESWAN balances clinical feasibility and research depth. Its multi-echo design, R2* quantification, and valence state sensitivity fill the technical gap between qualitative SWI and complex quantitative QSM.

At the clinical translation level, quantitative indicators of ESWAN (such as R2* and magnitude values) can serve as real-time parameters for efficacy monitoring. For example, the R2* average value in the treatment group decreased by 11.13% compared with the PD group in this study, which complemented the iron staining results and provided an imaging basis for optimizing probiotic administration protocols (such as dosage and course of treatment). In the future, dynamic contrast-enhanced MRI may be combined to further explore the correlation between iron metabolism and blood–brain barrier function.

### 4.3 Correlation between motor function recovery and iron deposition

Correlation analysis of behavior and imaging showed that the time that rats persisted on the wire was strongly positively correlated with the magnetic resonance magnitude value (*r* = 0.78, *p* < 0.001), indicating that the degree of iron deposition in the SN is closely related to motor coordination. Mechanistically, iron produces reactive oxygen species through the Fenton reaction, leading to lipid peroxidation and mitochondrial dysfunction, ultimately causing dopaminergic neuron death (Lin et al., 2025; Xiao et al., 2024; Zhou et al., 2024), resulting in motor dysfunction. Excessive iron deposition also has a synergistic effect with α-synuclein aggregation (Chen et al., 2025), and inhibits the activity of mitochondrial respiratory chain complexes (AlRuwaili et al., 2025; Duan et al., 2022), exacerbating neuronal energy metabolism disorders. After probiotic treatment, the partial recovery of motor function (the average value of time on wire in the treatment group was prolonged by 66.69% compared with the PD group) was consistent with the trend of reduced iron deposition, confirming the core role of iron metabolism regulation in PD pathology.

However, probiotic treatment failed to fully restore motor function to the control level, possibly due to two reasons: first, the degree of iron deposition reduction was insufficient to reverse the formed neuronal network damage; second, the impact of gut microbiota metabolites on motor cortex plasticity remains unclear, which is a direction worthy of exploration in the future.

### 4.4 Research limitations and future directions

This study has two limitations: first, the lack of long-term follow-up data makes it impossible to clarify the durability of the probiotic effect and the optimal administration cycle; second, due to the constraints of experimental conditions, the research on the mechanism of the neuroprotective effect of probiotics is not in-depth enough, and the specific probiotic strains or metabolites that play a role have not been identified. Future research can be carried out from two aspects: at the basic research level, molecular mechanism studies can be conducted, and metabolomics can be used to analyze the association between gut microbiota composition and brain iron deposition, with a focus on the cross-organ regulatory effects of metabolites such as short-chain fatty acids and proline; at the translational research level, based on the results of basic research, we will gradually carry out large-scale and long-term follow-up experiments to clarify the lasting impact of probiotics on the homeostasis of gut microbiota in PD patients. Meanwhile, we will pay attention to the potential interactions between probiotics and other drugs or food components during long-term use, and establish a comprehensive safety monitoring system, so as to provide solid evidence for the wide application of probiotics in clinical treatment.

In conclusion, this study confirmed that probiotics can reduce iron deposition in the SN of PD rats and improve motor function by regulating the gut microbiota, whereas the ESWAN sequence provides a reliable tool for non-invasively evaluating brain iron metabolism. This finding provides experimental evidence for the application of the “gut-brain axis” theory in PD and lays a foundation for the clinical development of probiotic adjuvant therapy strategies. In the future, combined with dynamic ESWAN monitoring, a multi-dimensional evaluation system of “gut microbiota–brain iron metabolism–motor function” can be constructed to promote the development of personalized PD treatment.

## Data Availability

The original contributions presented in this study are included in this article/supplementary material, further inquiries can be directed to the corresponding author.
